# Self-directed learning in veterinary medicine: are the students ready?

**DOI:** 10.5116/ijme.5929.402f

**Published:** 2017-06-14

**Authors:** Munashe Chigerwe, Karen A. Boudreaux, Jan E. Ilkiw

**Affiliations:** 1 Department of Veterinary Medicine and Epidemiology, University of California Davis, Davis, CA, USA; 2 Dean's Office, University of California Davis, Davis, CA, USA; 3 Department of Surgical and Radiological Sciences, University of California Davis, Davis, CA, USA

**Keywords:** Self-directed learning, veterinary medicine, medical students

## Introduction

Self-directed learning (SDL) is defined as a process or a learning method in which individuals take initiative, with or without the help of others, in diagnosing their needs, formulating goals, identifying human and material resources for learning, choosing and implementing appropriate learning strategies and evaluating outcomes.^1^ As medical knowledge continuously changes, health care professionals need to acquire skills that will enable them to be life-long learners. Self-directed learning (SDL) is considered one of the most appropriate learning method for health care professionals to keep up-to-date with current medical advances and literature.^2^ The North American Veterinary Medical Education Consortium considers SDL critical to life-long learning.^3^ Several veterinary schools, including the University of California Davis, School of Veterinary Medicine (UCDSVM) recently revised their veterinary curricula and incorporated learning approaches that foster SDL. The revised curriculum at UCDSVM incorporated inquiry-based and student-centered learning approaches that promote SDL, such as problem-based learning, case-based learning, team-based learning, and small-group learning. However, a significant proportion of instructors teaching in the veterinary curriculum at the UCDSVM had the perception that most veterinary students were likely unprepared for approaches that foster SDL, and suggested that most veterinary students preferred teacher-directed learning approaches, such as lectures. The instructors indicated that introduction of SDL would lead to an undesirable learning environment, and frustration by both students and instructors. The question posed at the time of implementation of the revised curriculum was; “Are the students prepared to learn veterinary medicine in a curriculum that incorporate methods that foster SDL?”

### Assessing SDL in veterinary students

Self-directed learning readiness was assessed prior to commencement of classes for students enrolled into the revised curriculum at the UCDSVM in 2016. An online SDL assessment tool which generated SDL readiness scores and their interpretation was performed for each student and the whole class.^4^

### Lessons learned

In contrast to the instructors’ perception, most students preferred learning approaches that foster SDL. Thus, the students were likely to perform well on tasks that require a high degree of problem solving skills, creativity and change. Another finding was that age, gender and educational background prior to enrollment into veterinary school were not significant predictors of SDL readiness scores. Although we did not specifically survey the instructors, we find their perception that students were not ready to learn in a curriculum that fosters SDL interesting. There is a potential conflict between students who prefer learning approaches that foster SDL and instructors who perceive students as not ready for SDL learning approaches. Is it possible that instructors were not familiar with some of the teaching methods that foster SDL, or they did not realize the value of SDL in the veterinary curriculum? Furthermore, did the instructors need specific training on teaching methods that foster SDL which they were not familiar with, or they needed to consider the value of SDL over time in the curriculum?

## Conclusions

Veterinary students prefer learning approaches that foster SDL which is desirable for the students’ long life learning. We anticipate that educators facilitating in the veterinary curricula will consider teaching methods that fosters SDL when presenting learning material. It should be noted that assessments of SDL in veterinary students should also assess instructor’s perceptions regarding SDL because instructors might perceive that students are not ready for learning approaches that foster SDL.

### Conflict of Interest

The authors declare that they have no conflict of interest.
